# Adipose/Connective Tissue From Thyroid-Associated Ophthalmopathy Uncovers Interdependence Between Methylation and Disease Pathogenesis: A Genome-Wide Methylation Analysis

**DOI:** 10.3389/fcell.2021.716871

**Published:** 2021-09-08

**Authors:** Yu Liang, Sijia Ding, Xiying Wang, Chunchun Hu, Yihan Zhang, Yan Hu, Yuye Zhang, Hongyu Kong, Weiyi Xia, Qinghe Jing, Yuxiang Hu, Chen Zhao, Lianqun Wu

**Affiliations:** ^1^Eye Institute and Department of Ophthalmology, Eye and ENT Hospital, Fudan University, Shanghai, China; ^2^NHC Key Laboratory of Myopia (Fudan University), Shanghai, China; ^3^Key Laboratory of Myopia, Chinese Academy of Medical Sciences, Shanghai, China; ^4^Shanghai Key Laboratory of Visual Impairment and Restoration, Shanghai, China; ^5^Department of Phase 1 Clinical Trial Unit, The First Affiliated Hospital with Nanjing Medical University, Nanjing, China

**Keywords:** thyroid-associated ophthalmopathy, methylation, orbital adipose/connective tissue, methylation array, pathology

## Abstract

In response to pathological stimulation, methylation status conversion of the genome drives changes of cell feature and is able to promote disease development. Yet the role of methylation in the development of thyroid-associated ophthalmopathy (TAO) remains to be evaluated. Overexpansion of orbital tissue is the key feature of TAO. In this study, the methylation profile of orbital adipose/connective tissue from TAO patients and normal individuals were compared. After screening 3,739 differentially methylated probes, the distribution and properties of these probes were analyzed. Furthermore, enriched biological functions of these genes associated with differential methylation and the relationship between their methylation status and expression profile were also identified, including PTPRU and VCAM-1. According to our results, methylation was involved in disregulated immune response and inflammation in TAO and might contribute to activation of fibroblast and adipogenesis, leading to the expansion of orbital tissue. Neuropathy and neurobehavioral symptoms were also potentially associated with methylation. These results may help to extend the understanding of methylation in TAO and provide more insights into diagnosis and treatment of patients.

## Introduction

Thyroid-associated ophthalmopathy (TAO) is a sight-threatening orbital disease and the leading type of autoimmune inflammatory disorder of the orbit (Li et al., 2018). Typical clinical features include orbital inflammation, exophthalmos, eyelid retraction, diplopia and eye movement limitation (Bartley and Gorman, 1995). In severe cases, vision can be compromised due to exposure keratitis, corneal ulcers and even compressive optic neuropathy (Bahn, 2010). Moreover, it cannot be ignored that patients with TAO may experience impaired quality of life and social function, and they could develop neurobehavioral syndrome in the form of anxious to depressive or psychotic disorders (Bruscolini et al., 2018). Therefore, TAO is a public health problem and its pathogenesis and treatment are worthy of further study.

To date, TAO is recognized as an autoimmune condition mostly associated with Graves’ disease (Bahn, 2010). In response to perturbed immune response shared by the orbit and thyroid, imbalanced B cell immunity and defected T cell subsets induced the phenotypic and functional changes in orbit (Fang et al., 2021). The activation of orbital fibroblast (OF) and process of adipogenesis are major factors contributing to overexpansion of retrobulbar space and clinical symptoms (Wang and Smith, 2014; Dik et al., 2016). Moreover, OF is the critical provider of autoantigens including thyroid stimulating hormone receptor (TSHR) and insulin-like growth factor-1 receptor (IGF-1R), suggesting that it can be both the instigator and main pathological effector of TAO (Smith, 2015). However, the pathogenesis of TAO is still unclear. A variety of gene polymorphisms related to TAO have been found, including TSHR, CTLA-4, CD40, HLA-DR, HLA-DQ, and TNF-α (Eckstein et al., 2009; Yang et al., 2017). Recently, the critical roles of environment and epigenetics in the development of TAO have been emphasized (Yin et al., 2012).

Epigenetics is one of the most rapidly expanding fields in biology (Portela and Esteller, 2010). DNA methylation is a major epigenetic mechanism involving the transfer of a methyl group onto the C5 position of the cytosine to form 5-methylcytosine (Moore et al., 2013). With breakthroughs in epigenetics research, it has been found that DNA methylation plays a prominent and dynamic role in regulating gene expression, which is involved in genetic imprinting (Li et al., 1993), embryonic development (Plass and Soloway, 2002; Smith and Meissner, 2013) and transcriptional regulation (Morales-Nebreda et al., 2019). In pathological conditions, differential methylation also orchestrates the expression of cytokine-associated genes and promotes the dysregulation of inflammation as well as autoimmune response (Surace and Hedrich, 2019). Given that DNA methylation often results from environmental factors and is reversible, it has been an important area in the study in molecular mechanism and therapeutic targets of several diseases, such as cancer (Dawson and Kouzarides, 2012), cardiovascular disorders (Castellani et al., 2020), thyroid disorders (Wang et al., 2017), and autoimmune disease (Wu et al., 2019). Moreover, aberrant DNA methylation has been reported to be highly associated with a range of ophthalmic diseases, including myopia (Seow et al., 2019), glaucoma (Johnson et al., 2018), cataract (Liu et al., 2020), age-related macular degeneration (Baird and Wei, 2013), and uveitis (Wei et al., 2020). In terms of TAO, although the DNA methylation profile of peripheral blood has been detected in TAO patients (Xin et al., 2019), research on the role of methylation modification in the pathogenesis of TAO is still needed.

In this study, using Illumina HD 850K methylation array, the genome-wide DNA methylation difference between orbital tissue from TAO patients and normal subjects was compared, followed by analysis of enriched function and biological processes (BPs). Afterward, integrative transcriptomics and DNA methylation analysis was conducted to further elucidate the interdependence between methylation and transcription. In addition, pyrosequencing was used to quantify DNA methylation level. Through detailed analysis, this study may provide more insights into the development of TAO and new targeted genes for treatment strategy.

## Materials and Methods

### Sample Collection and Preparation

Patients diagnosed with TAO with a clinical activity score less than 3 for more than 6 months were selected as participants in this study, according to the Bartley criteria (Bartley and Gorman, 1995; Mourits et al., 1997). TAO patients who met exclusion criteria, such as those having other inflammatory or autoimmune diseases or accepting radio-iodine therapy, steroids or immunosuppressive drugs within half a year before the study, were screened out. Adipose/connective tissue samples were obtained from involved TAO patients during orbital decompression surgery and from the control subjects during plastic operations. Control subjects with a history of thyroid, orbital diseases or any inflammatory/autoimmune disease were also excluded.

The above protocols are in compliance with the Declaration of Helsinki. Informed consent forms were provided to every patient before enrollment. This study was approved by the Ethics Committee of Changzheng Hospital, Second Military Medical University.

### Genome-Wide DNA Methylation Analysis

Total RNA was isolated from the samples of TAO patients and control subjects. Genome-wide DNA methylation was predominantly detected using the Illumina Infinium 850K human methylation assay, which includes 851,764 cytosine positions of the human genome covering > 14,000 genes. Genomic DNA was isolated from the samples using QIAamp DNA and Blood Mini Kits (QIAGEN, Hilden, Germany) and then bisulfite-converted using the Zymo EZ DNA Methylation Kit (Zymo Research, Irvine, CA, United States) according to the instruction manuals. Bisulfite-converted DNA was isothermally amplified and enzymatically fragmented, followed by purification and hybridization with the Infinium 850K array (Illumina, San Diego, CA, United States). The protocol included two bead types (Signal A for unmethylated alleles and Signal B for methylated alleles) for each CpG locus with the dual-color channel approach. Microarray data were extracted and the DNA methylation level was calculated using GenomeStudio Methylation Module v1.8 software (Version 2011.1, Illumina). Data were normalized by subtracting the background value, which was determined by an average of the signals of built-in negative control bead types. Methylation levels were compared between the TAO and control groups. The DNA methylation level of each interrogated CpG locus was determined by the β value through the following formula:

$$\beta =\frac{\text{Max}\,\left(\text{SignalB};0\right)}{\text{Max}\,\left(\text{SignalA},0\right)+\mathrm{Max}\,\left(\text{SignalB},0\right)+100}$$

The β value ranged from 0 to 1. Zero indicated complete demethylation and 1 indicated full methylation. Genes with positive DiffScores were then analyzed to determine which were the same.

### DMP Distribution Analysis

The differential methylated probes (DMPs) were stratified per genetic feature/chromosome and compared with the total number of 850k probes associated with the respective genetic feature/chromosome. In 850k annotation, CpG “island” is termed as a region in which the frequency of CG dinucleotides is higher than expectation. Genomic regions of 2,000 base pairs (2 kb) to each side of a CGI are CpG “shores,” with CpG “shelves” extending 2 kb beyond CpG shores and the rest of the genome termed as “open sea.” The significantly different methylation sites were screened by the *t*-test model, as defined by a threshold of |Δβ| > 0.17 and *P* < 0.05.

### Gene Ontology and Pathway-Enrichment Analysis

Differently methylated sites were used for unsupervised hierarchical clustering by the seaborn.clustermap in Python. Gene Ontology (GO)^1^ and Kyoto Encyclopedia of Genes and Genomes (KEGG)^2^ pathway enrichment analyses were performed using the Python scripts to clarify the function and biological pathways of differentially expressed methylation loci from our data. GO terms and KEGG analysis with *P* < 0.05 were considered significantly enriched by differential methylation loci-related genes.

### Validation by Pyrosequencing and Real-Time Quantitative RT-PCR

For pyrosequencing, DNA was firstly bisulfite-converted using the EpiTect Fast DNA Bisulfite Kit (QIAGEN) and then amplified by polymerase chain reaction (PCR) using the PyroMark PCR Kit (QIAGEN) in a total reaction volume of 25 μl that contained 50 ng DNA. Primers were designed using Pyrosequencing Assay Design Software (Biotage AB, Uppsala, Sweden). Sequences of the primers are listed in Supplementary Table 1. After purification, 20 μl PCR product was pyrosequenced using the PyroMark Gold Q96 Kit (QIAGEN) and PyroMark Gold Q96 pyrosequencer (QIAGEN) according to the manufacturer’s instructions. Data were collected and analyzed using PyroMark Q96 software (Version 2.5.8, QIAGEN). CpG methylation level (ranging from 0 to 1) was represented by the percentage of methylated C within the sum of methylated and unmethylated C.

For RT-PCR, Total RNA was extracted from samples using the TRIzol reagent (Invitrogen) according to the manufacturer’s specifications. Quantification was performed with reverse transcription (RT) and PCR processes. RT reactions in a total volume of 10 μl were implemented in a GeneAmp^®^ PCR System 9,700 (Applied Biosystems, United States) for 15 min at 42°C, 5 s at 85°C. Real-time PCR was performed using LightCycler^®^ 480 II Real-time PCR Instrument (Roche, Swiss) with 10 μl PCR reaction mixture. Reactions were incubated in a 384-well optical plate (Roche, Swiss) at 94°C for 30 s, followed by 45 cycles of 94°C for 5 s, 60°C for 30 s. The expression levels of mRNAs were normalized to ACTB and were calculated using the 2^–ΔΔCt^ method. Sequences of primers are listed in Supplementary Table 2.

## Results

### Characteristics of Differentially Methylated Probes in Orbital Tissue of TAO Patients and Controls

**FIGURE 1:**
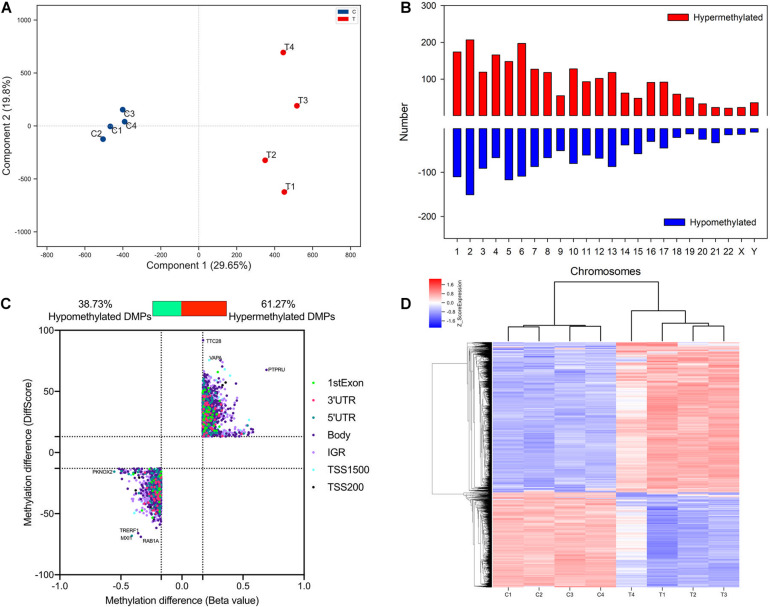
Identification and qualification of differentially methylated probes. **(A)** PCA analysis of methylation in TAO cases and controls. **(B)** Distribution of TAO-associated DMPs across chromosome. **(C)** Scatter plot of hyper- and hypo-DMPs based on threshold of | DiffScore| > 13 and | Beta value| > 0.17. **(D)** Heatmap of hierarchical clustering analysis for samples according to DMPs.

To detect altered methylation status in TAO patients compared with normal subjects, orbital adipose/connective tissues of TAO patients (*n* = 4, age 35.25 ± 6.34, male/female = 1/3) and control subjects (*n* = 4, age 39.00 ± 7.85, 4 female) were collected. The clinical activity scores of TAO patients were less than 3 and the mean duration of disease was 46.50 ± 7.89 months. There was no significant difference of age and gender between TAO and control subjects. Genome-wide methylation array was used to identify the methylation status of DNA in orbital tissue samples of TAO and control groups. PCA analysis indicated that the samples of two groups could be separated distinctly (Figure 1A). A total of 3739 DMPs were found across the genome in all chromosomes (Figure 1B).

Of 3,739 DMPs, hypermethylated DMPs accounted for 2,291, in which genes such as VAPA and PTPRU were significantly hypermethylated in TAO patients (Figure 1C). On the other hand, 1,448 DMPs were identified as hypomethylated probes, and differentially hypomethylated genes included TRERF1 and TFEC, etc. (Figure 1C). Top 15 hypermethylated- and hypomethylated probes and their regions were listed in Supplementary Tables 3, 4. Hierarchical clustering analysis revealed two distinct clusters, implying that the methylation status between TAO and control subjects could be clearly distinguished (Figure 1D).

**FIGURE 2:**
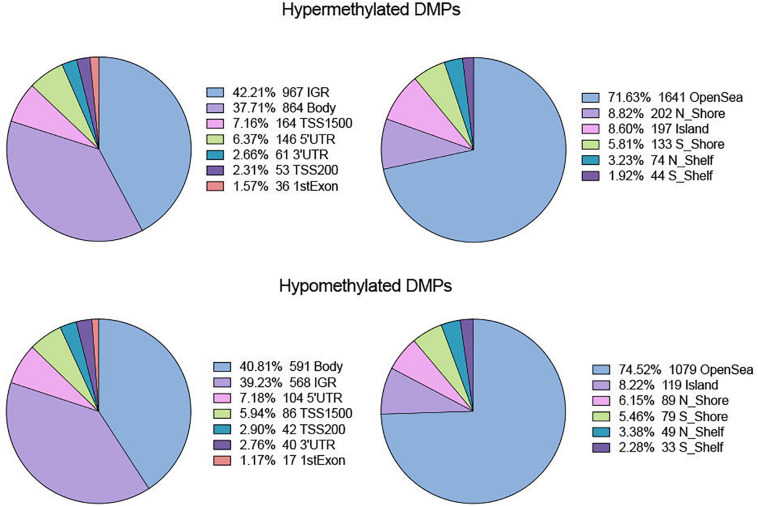
Distribution of hyper- and hypo-DMPs across different categories of genomic feature. Pie chart separately reveals the precentage of DMPs enriched in different gene regions (IGR, Body, TSS 1500, TSS200, 5′UTR, 3′UTR, and 1stExon) as well as in regions relative to CpG Island (OpenSea, N_Shore, S_Shore, N_Shelf, S_Shelf, and Island).

In terms of genomic location, there was no obvious difference in distribution between hyper- and hypomethylated DMPs (Figure 2). The majority of both DMPs were located in the gene body and intergenic region (IGR), while more than 70% of hyper- and hypomethylated DMPs were enriched in the open sea region relative to the CpG island. Located there were 8.82% of hypermethylated probes and 8.22% of hypomethylated probes.

### GO-Enriched Functional Analysis of DMPs

**FIGURE 3:**
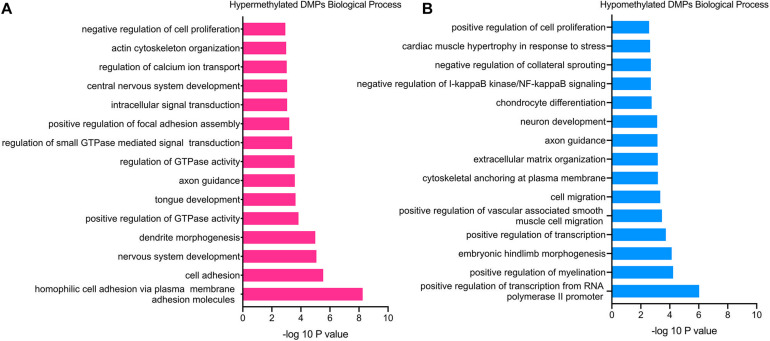
GO enrichment analysis of TAO-associated DMPs. **(A)** Top 15 most significant biological process (BP) terms of hypermethylated DMPs. **(B)** Top 15 most significant biological process (BP) terms of hypomethylated DMPs.

GO functional enrichment analysis revealed that the hypermethylated genes were closely related to cell proliferation and adhesion, including the BP category of negative regulation of cell proliferation (TGFB2, PTPRU, CDK6, etc.), homophilic cell adhesion via plasma membrane adhesion molecules (PCDHAC1, CDH13, SDK1, etc.), positive regulation of focal adhesion assembly (PAC1, ROCK1, LIMS1, etc.), actin cytoskeleton organization (ADD2, FGD4, ROCK1, etc.) and so on (Figure 3A). Besides, the hypermethylated genes were also associated with the nervous system, such as the BP of nervous system development (RBFOX1, NEUROG1, FGF14, etc.), axon guidance (BDNF, GLI3, TGFB2, etc.), and dendrite morphogenesis (RAC1, SDC2, LRP4, etc.).

**FIGURE 4:**
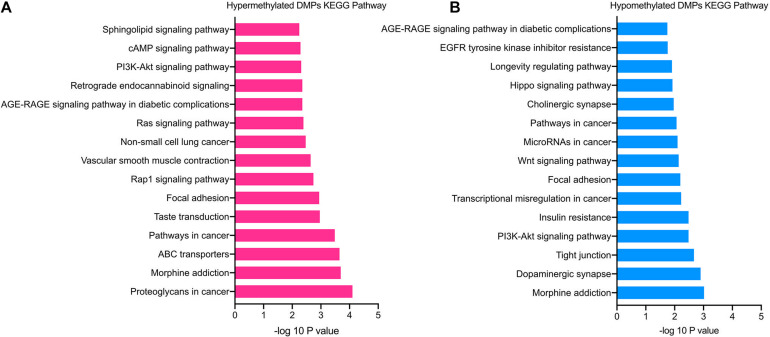
KEGG pathway enrichment analysis of TAO-associated DMPs. **(A)** Top 15 enriched KEGG pathways of hypermethylated DMPs. **(B)** Top 15 enriched KEGG pathways of hypomethylated DMPs.

In terms of hypomethylated genes, GO analysis demonstrated similar enriched BP as hypermethylated genes. The significantly enriched processes included the BP category of cell migration (RAB1A, PTEN, FOXC1, etc.), positive regulation of cell proliferation (GCNT2, HGF, FGFBP1, etc.), extracellular matrix organization (VCAM1, COL9A1, SMOC2, etc.), and negative regulation of I-kappaB kinase/NF-kappaB signaling (RIPK1, TLE1, TNIP3), which were all involved in the pathogenesis of TAO (Figure 3B). In addition, the BP of positive regulation of myelination (NRG1, HGF, PARD3, etc.), axon guidance (WNT3, NRP1, NCAM1, etc.), and neuron development (FIG4, MEF2C, GLI2, etc.) was enriched hypomethylated genes, which was still consistent with the enriched BP of hypermethylated genes. Furthermore, enriched BPs that might involve the overexpansion of adipose/connective tissue—for instance, BP of cardiac muscle hypertrophy in response to stress (GATA6 and MEF2C)—were detected.

### KEGG Analysis of DMPs

The significantly enriched pathways of hypermethylated DMPs included cAMP, PI3K-Akt, Rap1, and sphingolipid signaling pathways, focal adhesion, ABC transporters and morphine addiction (Figure 4A). Hypomethylated DMPs were significantly enriched in the Wnt signaling pathway, Hippo signaling pathway, tight junction and dopaminergic synapse (Figure 4B). In addition, the top 15 enriched KEGG pathways of hyper- and hypomethylated DMPs overlapped the signaling pathways of PI3K-Akt signaling pathway and focal adhesion. In accordance with GO analysis, hypomethylated DMPs were also enriched in pathways correlated with the nervous system, such as the dopaminergic synapse.

### Co-analysis of DMPs and Transcriptome Profile of Genes

**FIGURE 5:**
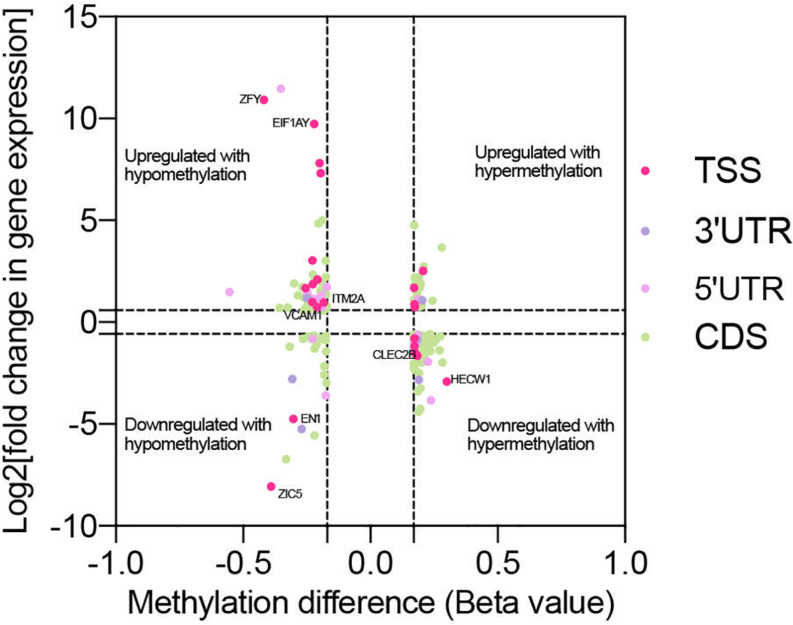
Co-analysis of genes with significant change in methylation and expression level. Scatter plot reveals the distribution of genes with differential methylation and expression. The genes were screened out with the threshold of | Beta value| > 0.17, log2| fold change| > 1 and *p* < 0.05. Based on the region of methylation, genes were labeled with different colors.

Genes with significant changes in both methylation and gene expression levels (with the thresholds of | Beta value| > 0.17, log2| fold change| > 1 and *p* < 0.05) were analyzed. The majority of the genes’ expressions were negatively related to methylation levels. It meant that the downregulated genes’ expression was associated with hypermethylation, and vice-versa (Figure 5). Moreover, methylation in different gene regions exerted varied influences on expression. Hypermethylation in TSS, also known as the promoter region, was associated with downregulated expression of most genes, including HECW1 and CLEC2B, while decreased methylation levels in this region were accompanied with increased expression of genes, including VCAM1 and ITM2A. The silencing function of methylation surrounding the promoter region is a well-known regulatory pattern on gene expression, and it is consistent with our results. However, the results also demonstrated that some genes, such as ZIC5 and EN1, were upregulated with promoter hypermethylation.

### Validation of DMPs

Based on genome-wide DNA methylation analysis and co-analysis, eight methylation sites on TTC28 (cg22359642), PTPRU (cg03627409), ACTN1 (cg14527649), RAB1A (cg00570635), VCAM1 (cg25763716), EIF1AY (cg26983535), CLEC2B (cg09947985), and PTPRQ (cg20238308) were selected for further validation.

**FIGURE 6:**
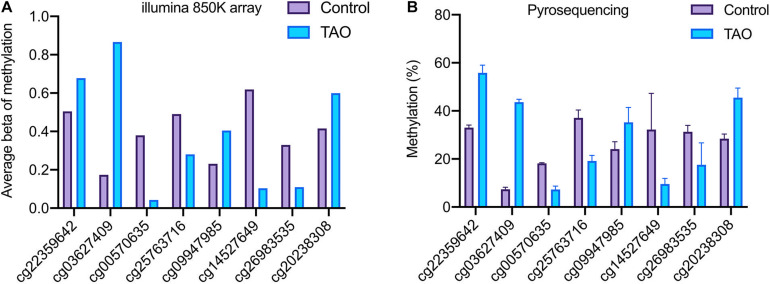
Validation of the methylation status of DMPs in TAO and control groups. **(A)** Methylation level of TTC28 (cg22359642), PTPRU (cg03627409), ACTN1 (cg14527649), RAB1A (cg00570635), VCAM1 (cg25763716), EIF1AY (cg26983535), CLEC2B (cg09947985), and PTPRQ (cg20238308) according to methylation array. **(B)** Methylation level of selected genes according to pyrosequencing.

It was noticed that pyrosequencing analysis revealed consistent consequences with the results of methylation array (Figure 6). The methylation levels of probe sites on TTC28, PTPRU, CLEC2B, and PTPRQ were upregulated, while sites on RAB1A, VCAM1, ACTN1, and EIF1AY were downregulated. Besides, eight genes (ZIC5, EN1, HECW1, CLEC2B, VCAM1, ITM2A, EIF1AY, and ZFY) with differential methylation and differential expression levels were also validated. In accordance with RNA-seq analysis, the expression levels of ZIC5, EN1, HECW1, and CLEC2B were decreased and the levels of VCAM1, ITM2A, EIF1AY, and ZFY were increased in TAO samples (Supplementary Figure 1).

## Discussion

Growing understanding of epigenetics has attracted more researchers to study its role on human diseases. DNA methylation is a stable but modifiable epigenetic process whereby a methyl group is transferred onto the C5 position of a cytosine on DNA sequences and regulates gene expression in response to environmental stimulation. Changed methylation levels are observed in the peripheral blood samples of TAO patients (Xin et al., 2019); however, its regulatory pattern in genome of orbital tissues remains to be evaluated.

In this study, we analyzed and compared the 850K methylation sites throughout the genome using the Illumina HD 850K methylation array in the orbital adipose/connective tissue of TAO patients and control subjects. A total of 3,739 DMPs were identified, with the majority of these located at the gene-body and intergenic regions, and at the open sea region relative to CpG island. The enriched GO of hyper- and hypomethylated genes shared similar functions, including cell adhesion, proliferation and migration, while their enriched pathways were associated with inflammation and nervous system in common. Previous study of function enrichment analysis in peripheral blood samples of TAO patients revealed that identified genes are involved with functions of axon guidance, focal adhesion and so on (Xin et al., 2019). Similar results are found in orbital tissue, and multiple unique functions were also exhibited in our study.

Perturbed immune response and inflammation are canonical mechanisms involved in TAO development. Several studies have proved that autoreactive inflammatory reaction can instigate the pathological process of OFs and almost all symptoms (Łacheta et al., 2019). Besides, abnormal immune activation is associated with the change of cell phenotypes and functions, such as macrophage polarization, which are also ascribed to the altered genomic methylation status (Wang et al., 2016). In our study, the results showed that methylation in adipose/connective tissue is involved in inflammation signaling mainly through the BP of negative regulation of I-kappaB kinase/NF-kappaB signaling and pathway of AGE-RAGE signaling pathway in diabetic complications. NF-kB mediated signaling is an acknowledged inflammatory process in diseases. Accumulated evidence suggests that DNA methylation participates in the activation of NF-kB inflammatory signaling transduction (Lu and Stark, 2015). The enrichment of methylation in genes associated with AGE-RAGE signaling pathways made us pay attention to the connection between inflammation, oxidative stress and advanced glycation end products (AGEs) in TAO. AGEs are a group of harmful glycated byproducts of the polyol pathway. Stimulated by hyperglycemia, AGE-RAGE signaling often plays a role in diabetic complications and has been also found to help regulate thyroid hormone secretion (Chen et al., 2019). In many disease models, activation of AGE-RAGE signaling increases levels of NF-kB and oxidative stress (Chen et al., 2017; Moura et al., 2020), implying its role in chronic inflammation. Thus, AGEs and their receptors may both participate in the development of TAO through the conversion of methylation status in genes related with those processes.

Besides, changed DNA methylation might contribute to the overexpansion of adipose/connective tissue. Increased volume of retrobulbar space is mainly ascribed to the hyperplasia of tissue and amplification of the extracellular matrix (Wang and Smith, 2014). In this study, the enriched BP related to positive regulation of cell proliferation, cell adhesion and cytoskeleton organization were all primordial mechanisms underlying the cell proliferation that might be involved in the activation and enlargement of adipose/connective tissue (Jones et al., 2019). The enriched BP of extracellular matrix organization suggested the alteration of ECM during volume amplification. In TAO, remodeling and overexpansion of ECM is often ascribed to OF by secreting glycosaminoglycans (GAGs) (Dik et al., 2016). GAG, particularly hyaluronan, is an important component in ECM that can regulate characteristics of microenvironment and absorb liquid to increase tissue volume (Hansen et al., 1997; Garantziotis and Savani, 2019). Previous studies reported that DNA methylation modulates hyaluronan metabolism, indirectly resulting in change of the ECM nature (Vigetti et al., 2014). Consequently, enriched BP of ECM organization may be also associated with the activation of OF and functions of GAGs. Furthermore, several differentially methylated genes, including HGF and CD44, is related to BP of hyaluronan metabolic process and catabolic process, implying indirect regulation of methylation on the process of hyaluronan production. It has been proven that the secretion of GAGs influences the proliferation, cellular signaling and adhesion of cells concentration-dependently in other cell models (Fotia et al., 2013; Zhao et al., 2015). The enriched BPs of cell adhesion and proliferation in function analysis may also connect with the production of GAGs in adipose/connective tissue.

Other signaling pathways that contribute to cell proliferation were also detected in KEGG analysis. The Hippo signaling pathway plays a critical role in regulating cell fates and organ growth by controlling cell proliferation and contacts (Misra and Irvine, 2018). Cross-talking with ECM, the Hippo signaling pathway could help activate fibroblasts and amplify pulmonary fibrosis (Liu et al., 2015), but its potential role in tissue remodeling and fibrosis of TAO is still poorly investigated.

Adipogenesis, which refers to the differentiation of peri-adipocytes, a type of fibroblast, into adipocytes and the generation of lipid, significantly contributes to hyperplasia of the orbital adipose tissue. It has been revealed that the inflammatory byproduct, AGEs, induce autophagy and accumulation of lipid in diabetic patients (Verma and Manna, 2016). On the other hand, another enriched pathway, the Wnt signaling pathway, helps constitute a regulatory network to adipogenesis (Christodoulides et al., 2009). Differential methylated ABCG1, which is associated with ABC transporters pathway, is also a verified regulator of adipogenesis (Khamis et al., 2020). Specific BPs, including cytoskeleton organization, are also implicated in the dynamics of adipogenesis in the development of TAO (Padilla-Benavides et al., 2016). The above results also suggested that the adipogenesis process in TAO is another main target modulated by methylation.

Given that methylation plays a general role in critical pathology processes, we can investigate genes with differential methylation as potential targets to study and interfere with TAO. Hence, several genes, including PTPRU and VCAM-1, were screened for future research. Consistent with previous results, these genes were mostly related to cell adhesion, differentiation, immune response or adipogenesis.

Protein tyrosine phosphatase receptor U (PTPRU), as a controller of cell phosphotyroine level to regulate cell–cell signaling, was one of the most hypermethylated genes on its gene body region. Previous studies indicated that PTPRU modulated cell adhesion and proliferation through the Wnt/β-catenin signaling pathway (Yan et al., 2006). Wnt signaling pathway was validated to interfere with adipogenesis process by inhibiting PPARγ and C/EBPα (Ross et al., 2000). Thus, PTPRU might influence the tyrosine phosphorylation of β-catenin and downstream signaling to indirectly regulate adipogenesis-related factors. In gastroenteric cancer, Hippo/YAP signaling transduction is also inhibited by PTPRU, leading to the attenuation of cancer (Gu et al., 2019). Thus, regulation of methylation level on PTPRU may influence adipogenesis and cell proliferation, modulating the development of TAO.

Based on our results, the expression level of vascular cell adhesion molecule-1 (VCAM-1) was upregulated parallel with hypomethylation. VCAM-1 exerts pro-inflammatory function in many autoimmune diseases and helps the migration and adhesion of macrophages and T cells to tissue (Kong et al., 2018). In TAO patients’ retrobulbar space, elevated VCAM-1 in endothelial cells has been detected to induce the recruitment of lymphocytes (Heufelder and Scriba, 1996). Except for endothelial cells, fibroblasts can express VCAM-1 in response to TNF-a, which has been proved in autoimmune rheumatoid arthritis (Lowin et al., 2020). In TAO patients, VCAM-1 can also be elevated by activated immune cells, such as Th17 cells, and mediates orbital fibrosis and adipogenesis (Fang et al., 2017, 2021). According to GO terms and KEGG analysis, VCAM-1 in the adipose/connective tissue of TAO patients was associated with immune response and the NF-kB signaling pathway. Consequently, VCAM-1 may contribute substantially to TAO, and methylation is an important mechanism to regulate it.

Additionally, out of our expectation, the results revealed significantly enriched functions of nervous development (e.g., regulation of myelination), and neural activity (e.g., morphine addiction, and dopaminergic synapse). These results can be explained by the influence of disregulation of thyroid hormone. In normal conditions, thyroid hormone show the ability of regulating neural cell proliferation, migration and myelination, as well as protecting neuroglial cells from pathological death in the central nervous system (Hartley et al., 2019; Chaudhary et al., 2020). In TAO patients, increased thyroid hormone can induce damage of neuron and glial cells, disrupting cell functions and the homeostasis of dopaminergic neuron activity, which might lead to neural complications (Atterwill et al., 1984; Cheng et al., 2010). In the I^131^-treated thyroid function fluctuated rat model, TAO is induced and swelling nerve fibers and shedding myelin can also be detected (Tu et al., 2016). Within these pathways, methylation in the gene body of neuregulin 1 (NRG1) was involved. NRG1 is a member of the neuregulin family that interacts with the EGFR family and modulates the repair of neural damage as well as immune response in the nervous system (Kataria et al., 2019). Apart from neural modulatory function, secretion of NRG1 from fibroblast and endothelial cells also participates in the fibrotic remodeling of myocardial cells (Dugaucquier et al., 2020). Whole-genome sequence analysis has revealed the association between NRG1 and free thyroxine (Taylor et al., 2015), while it has also been found to influence the predisposition to thyroid carcinoma (He et al., 2018). Thus, methylation of NRG1 may be one of the potential factors in the development of TAO.

We have performed gene expression analysis in TAO patients in previous article (Wu et al., 2021). Methylation analysis and expression analysis shared several identical results in GO terms and KEGG pathways. For instance, extracellular matrix organization (GO:0030198) is enriched in GO terms of hypomethylated genes and upregulated transcripts, while cell adhesion (GO:0007155) is enriched in hypermethylated and downregulated transcripts. In addition, Wnt signaling pathway (hsa04310), ABC transporters (hsa02010), PI3K-Akt signaling pathway (hsa04151), and focal adhesion (hsa04510), as important participants in TAO, are common enriched pathways in the two analyses, revealing latent correlation between methylation and gene expression. However, DNA methylation is reversible, and diverse patterns of methylation regulation have been detected, indicating that the consequence of methylation on specific genes can be variable. In combined analysis, we noticed that the expression profile of genes with hyer- or hypomethylation can be diverse. For example, promoters on ZIC5 and EN1 were hypermethylated, yet their expression level was decreased in TAO. This was partly explained by the site-dependent influence of methylation. Traditionally, methylation in promoter was a dominant factor associated with expression silencing. Nevertheless, merging research uncovered that gene body methylation is positively correlated with expression (Yang et al., 2014), which is possibly mediated by increased disruption of chromatin structure after decreased gene body modification (Yang et al., 2014). Other studies focusing on methylation of first exon revealed it is more tightly linked to downregulation of genes than the promoter region (Brenet et al., 2011). Taken together, the methylated site must be taken into consideration when analyzing the function of methylation in detail. However, we also found that methylation on the same region of different genes associated with altered the expression profile. We speculated that this might have resulted from the combined influence of methylation and other modifications. For example, there is a cross-talk between DNA methylation and histone modification, mediated by the interaction between the domain of histone methyltransferases and DNA methyltransfrases (Cedar and Bergman, 2009). DNA methylation also accounts for the production of miRNAs, leading to a complex regulatory network of gene expression (Fuso et al., 2020). Besides, according to differential DNA sequences, excess methylation can induce differential change in DNA secondary structures, which will influence the availability of DNA for transcription (Proctor et al., 2020).

On the other hand, differential methylation can be directly induced by pathological stimulation of TAO or a feedback protective response against TAO. The functions and specific role of differential methylated genes remain for further study. In addition, the orbital adipose/connective tissue of subjects was formed by diverse types of cells. This was involved with distinct methylation profiles in response to the same activation. Future analysis based on single cell sequencing of orbital tissue will provide more useful information.

As far as we know, our study is the first to examine the methylation profile of adipose/connective tissue in TAO patients compared with normal subjects. We demonstrated the characteristics of methylation status within the whole genome and screened differential methylated genes, contributing to autoimmune stimulation, overexpansion of tissue, adipogenesis and nervous response to pathological process. This analysis provided novel ideas about the role of methylation modification in the development of TAO. In those differentially methylated genes, PTPRU and VCAM-1 performed significant alteration in methylation level and potentially participated in pathogenesis. Those targeted genes all have potential for further research on underlying mechanism, diagnosis and therapeutic strategy.

## Data Availability Statement

The datasets presented in this study can be found in online repositories. The names of the repository/repositories and accession number(s) can be found below: https://www.ncbi.nlm.nih.gov/, GSE175399.

## Ethics Statement

The studies involving human participants were reviewed and approved by the Ethics Committee of Changzheng Hospital, Second Military Medical University. The patients/participants provided their written informed consent to participate in this study.

## Author Contributions

LW conceived the experiments and wrote the manuscript. YL drafted the manuscript and performed the experiments with assistance from SD and XW. YH, YYZ, and YXH collected the samples. CH, YHZ, HK, WX, and QJ analyzed the data. CZ and LW supervised the progress of the project and revised the manuscript. All authors read and approved the final submission.

## Conflict of Interest

The authors declare that the research was conducted in the absence of any commercial or financial relationships that could be construed as a potential conflict of interest.

## Publisher’s Note

All claims expressed in this article are solely those of the authors and do not necessarily represent those of their affiliated organizations, or those of the publisher, the editors and the reviewers. Any product that may be evaluated in this article, or claim that may be made by its manufacturer, is not guaranteed or endorsed by the publisher.

## Footnotes

1
http://www.geneontology.org

2
http://www.kegg.jp/
