# Supplementary material for: Adipose/Connective Tissue From Thyroid-Associated Ophthalmopathy Uncovers Interdependence Between Methylation and Disease Pathogenesis: A Genome-Wide Methylation Analysis
Source: Front Cell Dev Biol. 2021 Sep 8;9:716871. doi: 10.3389/fcell.2021.716871 (PMC8457400; doi:10.3389/fcell.2021.716871)
Supplement: Supplementary file 1 [file Table_1.DOCX]

**Supplementary Table 1.** Primers for pyrosequencing analysis

| TargetID | Symbol | Strand | region | Pyroseq Sequence (5'-3') |
| --- | --- | --- | --- | --- |
| cg22359642 | TTC28 | + | GeneBody | TTC28_F: TTTATGGGTATTAGGAAGAAGAATAGATT TTC28_R: CACCAAATCATATATATCTTTCACATAACA TTC28_SeqF: CACAAATCCAATACTCAACAATTC |
| cg03627409 | PTPRU | + | GeneBody | PTPRU_F: TGGGGAAGTTGAAGTTTTAAGGTA PTPRU_R: AACCTTTCCCTAAATTCCTACAAATTC PTPRU_SeqF: TGTGTAGAATAGAAAATTGTG |
| cg14527649 | ACTN1 | + | GeneBody | ACTN1_F: TTGATTGGTGGTGGTGAGGT ACTN1_R: CCACCCCAAAAAACATCTCATT ACTN1_SeqF: TTAGGTTTTGGGGAGA |
| cg00570635 | RAB1A | + | GeneBody | RAB1A_F: TGTTGGGATTATTGAGGTGAGTTA RAB1A_R: CAACCTCTAAATTTTTTTTCCCTTTTAT RAB1A_SeqF: AATGATGTATATTATTTGAAGAA |
| cg25763716 | VCAM1 | + | TSS1500 | VCAM1_F: ATGAATAAGAATTGGTTTTGATTATGAGA VCAM1_R: ACATAACCCAAAACCTTACAAAATACTT VCAM1_SeqF: AATGGGGGAGATAGA |
| cg26983535 | EIF1AY | + | TSS200 | EIF1AY_F: TAGGTGGGTGAGTAGAAGAGGTTGGTTATA EIF1AY_R: ATCCTTAACTCCACCTTCTCCATAC EIF1AY_SeqF: GTATTTTTTGGAGTTATTAAAAGG |
| cg09947985 | CLEC2B | + | TSS1500 | CLEC2B_F: AGTATGGTTGAGTGAAATTTAGTT CLEC2B_R: ACATCAAACTTACAAAAATTACCAACTAT CLEC2B_SeqF: AATATAGGGAAATGTT |
| cg20238308 | PTPRQ | + | TSS1500 | PTPRQ_F: AGGAAGAGAGATTATGAAGTTAAGT PTPRQ_R: TCCCTAAAACACCAAAAAAATAACC PTPRQ_SeqF: ACATTTTATTATATCCATTTTCAA |
