# Supplementary material for: Adipose/Connective Tissue From Thyroid-Associated Ophthalmopathy Uncovers Interdependence Between Methylation and Disease Pathogenesis: A Genome-Wide Methylation Analysis
Source: Front Cell Dev Biol. 2021 Sep 8;9:716871. doi: 10.3389/fcell.2021.716871 (PMC8457400; doi:10.3389/fcell.2021.716871)
Supplement: Supplementary file 2 [file Table_2.DOCX]

| **Gene Symbol** | **Forward primer (5'-3')** | **Reverse primer (5'-3')** | **Product length(bp)** | **Tm(℃)** |
| --- | --- | --- | --- | --- |
| ZFY | GTTATTGAATCGCCACCTCT | GGTGTCGGAAACCTTTACC | 83 | 60 |
| EIF1AY | AGAGAGTTGGTGTTTAAAGAGG | AAACACAATGCTTCCAATCG | 86 | 60 |
| ITM2A | AATAACAGAAAGTCCTTCCGC | CTTCCAGCATTTATCAATGGC | 81 | 60 |
| VCAM1 | ACCAAGAGTTTGGAAGTAACC | CAATGTGTAATTTAGCTCGGC | 82 | 60 |
| CLEC2B | GATCACTGGATTGGACTGAAG | TCCCTCTCATGCCAAACG | 91 | 60 |
| EN1 | AAGAAAGCCACAGGCATC | TACTCGCTCTCGTCTTTGTC | 104 | 60 |
| ZIC5 | ATAGCAGTGATCGGAAGAAAC | GGATTTGTCACAGCCTCG | 83 | 60 |
| HECW1 | GCCAAGCTCCGCAATTTCTA | ATGATCCCGGCGAATAATG | 90 | 60 |
| ACTB | CATTCCAAATATGAGATGCGTT | TACACGAAAGCAATGCTATCAC | 133 | 60 |

**Supplementary Table 2.** Primer sequences for quantitative real-time polymerase chain reaction (qRT-PCR) analysis.
