# Supplementary material for: Adipose/Connective Tissue From Thyroid-Associated Ophthalmopathy Uncovers Interdependence Between Methylation and Disease Pathogenesis: A Genome-Wide Methylation Analysis
Source: Front Cell Dev Biol. 2021 Sep 8;9:716871. doi: 10.3389/fcell.2021.716871 (PMC8457400; doi:10.3389/fcell.2021.716871)
Supplement: Supplementary file 3 [file Table_3.DOCX]

**Supplementary Table 3.** Top 15 hypermethylated probes in orbital adipose/connective tissues between TAO patients and control subjects

| Target ID | Annotated Gene | Gene Feature | Region related to CpG Island | Methylation Difference |
| --- | --- | --- | --- | --- |
| cg22359642 | TTC28 | Body | OpenSea | 91.79155 |
| cg01075918 | VAPA | TSS1500 | N_Shore | 75.69418 |
| cg10776061 | MAN2B1 | Body | S_Shore | 75.39874 |
| cg05210213 | / | IGR | OpenSea | 73.88956 |
| cg03187614 | TRIM10 | Body | OpenSea | 70.43449 |
| cg14027161 | VAPA | TSS1500 | N_Shore | 68.14484 |
| cg03627409 | PTPRU | Body | OpenSea | 67.59905 |
| cg06357842 | / | IGR | OpenSea | 66.41286 |
| cg09723088 | HLA-F | 1stExon | Island | 65.86136 |
| cg18648613 | PPP3CA | Body | OpenSea | 64.27875 |
| cg17419475 | WWC2 | Body | OpenSea | 63.66614 |
| cg01493038 | NRCAM | Body | OpenSea | 63.36697 |
| cg08624915 | AHSP | TSS1500 | OpenSea | 62.9832 |
| cg04011266 | CDKN2B-AS1 | Body | OpenSea | 62.84783 |
| cg18210083 | / | IGR | OpenSea | 62.50027 |
