# Supplementary material for: Adipose/Connective Tissue From Thyroid-Associated Ophthalmopathy Uncovers Interdependence Between Methylation and Disease Pathogenesis: A Genome-Wide Methylation Analysis
Source: Front Cell Dev Biol. 2021 Sep 8;9:716871. doi: 10.3389/fcell.2021.716871 (PMC8457400; doi:10.3389/fcell.2021.716871)
Supplement: Supplementary file 4 [file Table_4.DOCX]

**Supplementary Table 4.** Top 15 hypomethylated probes in orbital adipose/connective tissues between TAO patients and control subjects

| Target ID | Annotated Gene | Gene Feature | Region related to CpG Island | Methylation Difference |
| --- | --- | --- | --- | --- |
| cg00570635 | RAB1A | Body | N_Shore | -69.00044 |
| cg08690692 | TRERF1 | 5'UTR | OpenSea | -68.14877 |
| cg14609104 | MXI1 | Body | S_Shelf | -66.03963 |
| cg01710903 | RBPMS | Body | S_Shore | -61.7008 |
| cg09725852 | ROR1 | Body | OpenSea | -58.94688 |
| cg11787952 | / | IGR | OpenSea | -58.05145 |
| cg01425218 | / | IGR | N_Shore | -56.67562 |
| cg26770787 | PPP3CA | Body | OpenSea | -56.20906 |
| cg22411949 | / | IGR | OpenSea | -55.87762 |
| cg23700338 | EIF4G3 | Body | OpenSea | -55.8644 |
| cg02721176 | C10orf96 | Body | OpenSea | -55.47427 |
| cg03575050 | / | IGR | OpenSea | -55.09569 |
| cg04663487 | TFEC | TSS200 | OpenSea | -55.05095 |
| cg14604519 | RASSF3 | Body | OpenSea | -54.46783 |
| cg20854480 | SCHIP1 | Body | OpenSea | -54.45101 |
