# Supplementary material for: Adipose/Connective Tissue From Thyroid-Associated Ophthalmopathy Uncovers Interdependence Between Methylation and Disease Pathogenesis: A Genome-Wide Methylation Analysis
Source: Front Cell Dev Biol. 2021 Sep 8;9:716871. doi: 10.3389/fcell.2021.716871 (PMC8457400; doi:10.3389/fcell.2021.716871)
Supplement: Supplementary file 5 [file Table_5.DOCX]

Supplementary Material

**Supplementary Figure 1.** Validation of the expression pattern of genes with differential methylation and expression between TAO and control groups.
